# Editorial: Calcium Homeostasis in Skeletal Muscle Function, Plasticity, and Disease

**DOI:** 10.3389/fphys.2021.671292

**Published:** 2021-03-26

**Authors:** Matias Mosqueira, Heinrich Brinkmeier, Enrique Jaimovich

**Affiliations:** ^1^Institute of Physiology and Pathophysiology, Heidelberg University Hospital, Heidelberg, Germany; ^2^Institute of Pathophysiology, University Medical Center Greifswald, Karlsburg, Germany; ^3^Center for Exercise, Metabolism and Cancer Studies, Faculty of Medicine, University of Chile, Santiago, Chile

**Keywords:** exercise, SOCE, ROS, MCU, Stac3 protein, MG53 (or TRIM72), mechanical ventilation, tissue engineering

The pivotal discovery of calcium as the only ion able to produce muscle contraction was made by Lewis Victor Heilbrunn in 1947. Since then, the role of calcium in the skeletal muscle has been expanded and clarified as the essential protagonist of intracellular signaling activity, metabolism, tissue formation, maturation, and regeneration. Physiological and biochemical effects of calcium are translated into cellular functions by the activity of calcium-binding proteins. Pathological conditions alter calcium's physiological role, making calcium a central target of therapeutic strategies. This Research Topic entitled “Calcium Homeostasis in Skeletal Muscle Function, Plasticity and Disease” collected new and relevant information on the role of calcium in skeletal muscle aiming to establish a new point of reference for future muscle research. For instance, Mijares et al. investigated the association of senescence with elevated intracellular resting [Ca^2+^] in murine isolated single flexor digitorum brevis skeletal muscle fibers in parallel with an *in vivo* study. Using fluorescent ROS sensor CM-H2DCFDA in young (3 months-old), middle-aged (12 months-old), and aged (24 months-old) mice, they found an age-related increase in [Ca^2+^]_i_. When flufenamic acid, a non-steroidal anti-inflammatory was administered for several weeks the fluorescence levels were reduced in middle-aged and aged muscle fibers. This decrease was associated with a significant reduction of [Ca^2+^]_i_ as well as [Na^+^]_i_ and other pro-inflammatory markers.

It is known that Ca^2+^ plays a multifaceted role in mitochondrial function. During muscle contraction, Ca^2+^ influx into mitochondria activates multiple enzymes related to the tricarboxylic acid cycle and oxidative phosphorylation, resulting in increased ATP synthesis to meet the energy demand. Li et al. revised interesting mechanisms of how physiological Ca^2+^ transients vs. pathological steady-state Ca^2+^ elevation cause ROS increase in skeletal muscle mitochondria. Pathophysiological conditions such as skeletal muscle denervation or unloading also lead to elevated Ca^2+^ levels inside mitochondria. The outcomes of this steady-state elevation of mitochondrial Ca^2+^ level include exacerbated ROS generation, sensitized opening of mitochondrial permeability transition pore, induction of programmed cell death and ultimately muscle atrophy. However, both acute and long-term endurance exercise and electrical stimulation activate certain signaling pathways to counteract ROS production preventing apoptosis and alleviate muscle atrophy in denervated animal models and patients with motor impairment.

In this regard, Quezada et al. describe a new mechanism to explain the role of transient cytosolic Ca^2+^ signals and signaling pathways related to muscle plasticity by regulation of gene expression of the MCU complex in adult skeletal muscle. The Authors report that the MCU complex can be regulated by electrical stimuli in a frequency-dependent manner. The changes observed in mRNA levels may be related to changes in the mitochondria, due to phenotypic transition from a fast to a slow muscle type. Exogenous ATP decreases the mRNA levels of the MCU complex while MCU levels increase when basal [ATP] is reduced, indicating that extracellular ATP may be a regulator of the MCU complex and part of the axes linking low-frequency stimulation with ATP/IP3/IP3R.

Store-operated calcium entry (SOCE) is a fast mechanism responsible for replenish SR with Ca^2+^ that is controlled by SR-located STIM1 and Orai1 present in the sarcolemma. In rat skeletal muscle, but not in mouse, a phasic SOCE can be activated upon single action potentials. Lilliu et al. demonstrating that pSOCE can be electrically triggered in EDL skinned murine muscle after 5–6 days of voluntary wheel-running successfully answered this issue. This simple strategy expands the use of genetically modified mouse models to further comprehending the physiology and pathophysiology of SOCE.

A second paper related to Orai1 has been published in this Research Topic, focusing on cell physiological consequences of reduced Orai1 gene expression. Sztretye et al. used two models, muscles from myostatin deficient mice and muscles from WT mice, gene silenced for Orai1. The authors present changes in mitochondrial function, altered ultrastructure of the neuromuscular junction and reduced postsynaptic Ca^2+^ transients. They suggest that reduced Orai1 gene expression may be related to certain types of muscle weakness and alleviated neuromuscular transmission.

Silva-Rojas et al. reviewed the remarkable diversity of phenotypes and disorders related to STIM/Orai proteins. STIM1 and Orai1 are required for the regulation and fine-tuning of the Ca^2+^ level in the sarco-/endoplasmic reticulum of many cell types. Both, loss of function and gain of function of either protein cause alterations of cellular functions leading to diseases. Early data on loss of function of Orai1 reported immune deficiency and muscle weakness in children. Recent findings revealed dysfunctions of eye movement, skin, bone and spleen abnormalities as well as blood coagulation defects due to mutations in the STIM1 and Orai1 genes.

Elementary calcium release events known as Ca^2+^-sparks are observed in mammalian and non-mammalian skeletal muscle, characterizing the morphology and frequency of spontaneous Ca^2+^ release events. As in mammals, frogs also express two types of RyR. The α isoform is associated with DHPRs, while the β isoform is not connected with DHPR but sensitive to [Ca^2+^]_i_. Recording Ca^2+^-sparks from isolated frog fibers at high-speed acquisition (15.4 μs/line), Szappanos et al. show that caffeine-dependent Ca^2+^-spark are significantly larger and more frequently than the voltage-dependent. This result revealed the role of RyRβ in the generation of spontaneous Ca^2+^-sparks and shedding light on the interaction of RyRα and RyRβ during Ca^2+^ release.

Tubular aggregates (TAs) are characterized by abnormal accumulation of packed SR tubes, a histopathological feature in TA myopathy (TAM). TAM is linked to gain-of-function mutations in both STIM1 and Orai1 and is commonly found in human muscle disorders, such as dyskalemia, periodic paralysis, or myotonic disorders. Boncompagni et al. evaluated the presence of TAs, STIM1-Orai1 localization and expression and fatigue resistance in intact wild-type murine EDL muscles at 4-month-old, aged (24-month-old) and in wheel-running trained for 15 months (starting from 9 months-old). Based on the evidence that long-term exercise significantly reduced aged-dependent TAs formation and accumulation of STIM1 and Orai1 in TAs and exercise restored the capability of aged EDL to use external Ca^2+^, the Authors concluded that exercise maintains correct SOCE activity during aging.

New evidence of Ca^2+^ release's function during exercise is presented by Gejl et al. The Authors measured Ca^2+^ release from SR vesicles from muscles triceps brachii and vastus lateralis obtained from cross-country skiers and triathletes together with cyclists athletes 4-min single-bout of high-intensity exercise. The Authors showed reduced SR Ca^2+^ release after acute high-intensity training, which was further reduced when the athletes repeated the high-intensity exercise, without alteration on SERCA1 function. Together, this study demonstrated that short duration of high-intensity exercise adapts the EC-coupling reducing Ca^2+^ release without modifying Ca^2+^-uptake.

In vertebrates, Stac3 protein is responsible for DHPR's function and localization. Mutants of Stac3 showed significant reduction in muscle function due to dysfunction and mis-localization of DHPR. Hsu et al. demonstrated in larvae of Drosophila that the Stac3 (Dstac) expression pattern correlated with the DHPR's pattern in the T-tubule and Stac3 knockout resulted in a significantly disarranged DHPR localization, but no alteration in the T-tubule disposition. Consequently, larval locomotion was significantly affected due to reduced Ca^2+^ transients. These results, as seen in vertebrates, suggest that Stac3 is relevant for the normal EC-coupling in skeletal muscles.

Benissan-Messan et al. revised the pivotal function of Mitsugumin-53 (MG53), a protein from tripartite motif (TRIM) family responsible for EC-coupling function such as enhancing Ca^2+^-entry via SOCE and reducing RyR1 and SERCA activities as well at transcriptional level increasing TRPC3 and TRPC4 expressions and repair facilitating vesicle translocation to the plasma membrane after injury. MG53 also plays pivotal role on regeneration in different tissues besides skeletal muscle, resulting in an interesting key protein to be analyzed in several muscular dystrophies and aging.

Diaphragm' of patients under long-term mechanical ventilation (MV) reduce protein anabolism and increase protein catabolism, inducing diaphragmatic atrophy. This mechanism is known as ventilator-induced diaphragm dysfunction (VIDD). Hyatt and Powers reviewed new evidence of how mitochondrial ROS production during MV oxidates RyR1, leading to disassociation of calstabin1 from RyR1, resulting in Ca^2+^ leakage from the SR. Subsequently, high cytosolic Ca^2+^ triggers several proteolytic systems, which among them, calcium-activated protease calpain signaling pathway is the main responsible for VIDD. Therefore, it is relevant to control ROS production and re-establish Ca^2+^ homeostasis in the diaphragm during MV.

An interesting review by Khodabukus analyses the current evidence on tissue-engineered skeletal muscle models to study muscle function, plasticity, and disease; although small animal models have been essential for elucidating the molecular mechanisms regulating skeletal muscle adaptation and plasticity, these models do not always accurately model human muscle disease. The potential of *in vitro* three-dimensional tissue-engineered skeletal muscle models is discussed, as well as the genetic, neural, and hormonal factors regulating skeletal muscle fiber-type *in vivo* and the ability of current *in vitro* models to study muscle fiber-type regulation.

In summary, this Research Topic highlighted the most recent function of Calcium in skeletal muscle, covering from the molecular level through signaling pathways up to the whole body with innovative models giving relevant information for physiological and pathophysiological conditions.

## Author Contributions

All authors listed have made a substantial, direct and intellectual contribution to the work, and approved it for publication.

## Conflict of Interest

The authors declare that the research was conducted in the absence of any commercial or financial relationships that could be construed as a potential conflict of interest.

